# Comparison of a new gasless method and the conventional CO_2_ pneumoperitoneum method in laparoendoscopic single-site cholecystectomy: a prospective randomized clinical trial

**DOI:** 10.1007/s13304-021-01154-9

**Published:** 2021-08-31

**Authors:** Min Jiang, Gang Zhao, Anhua Huang, Kai Zhang, Bo Wang, Zhaoyan Jiang, Kan Ding, Hai Hu

**Affiliations:** Center of Gallstone Disease, Shanghai East Hospital, Tongji University School of Medicine, Shanghai, China

**Keywords:** Gasless laparoscopic surgery, Laparoendoscopic single-site surgery, Laparoscopic cholecystectomy, Carbon dioxide pneumoperitoneum, Randomized controlled trial

## Abstract

To avoid CO_2_ pneumoperitoneum-associated cardiopulmonary side-effects during conventional laparoscopic surgeries, we have developed a gasless laparoscopic operation field formation (LOFF) device for laparoendoscopic single-site surgery. The aim of this study is to analyze the safety and efficacy of the LOFF device for laparoendoscopic single-site cholecystectomy and to verify its advantage of avoiding CO_2_ pneumoperitoneum-associated complications. In this prospective, randomized, observer-blinded clinical trial, eligible participants were randomized in a 1:1 ratio to undergo either conventional CO_2_ pneumoperitoneum assisted laparoendoscopic single-site cholecystectomy (LESS) or the new gasless LOFF device assisted laparoendoscopic single-site cholecystectomy (LOFF-LESS). Outcomes including intra-operative respiratory and hemodynamic parameters, operation time, conversion rate, complication rate, et al were compared between the two groups. A total of 100 patients were randomized to the LESS group [*n* = 50; mean (SD) age, 49.5 (13.9) years; 24 (48.0%) women] and the LOFF-LESS group [*n* = 50, mean (SD) age, 47.4 (13.3) years; 27 (54.0%) women]. Compared with the LOFF-LESS group, the LESS group witnessed significant fluctuations in intra-operative respiratory and hemodynamic parameters. The tracheal extubation time of the LESS group was significantly longer (*P* = 0.001). The gasless LOFF device is safe and feasible for simple laparoscopic cholecystectomy and has a predominance of avoiding CO_2_ pneumoperitoneum-associated cardiopulmonary side-effects. Trial registration number: ChiCTR2000033702

**Graphic abstract:**

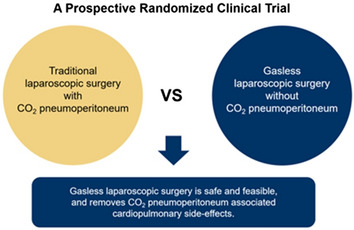

**Supplementary Information:**

The online version contains supplementary material available at 10.1007/s13304-021-01154-9.

## Introduction

The prerequisite of conventional laparoscopic surgery is the establishment of a clear operative space through a small incision in the abdominal wall. Conventionally, CO_2_ pneumoperitoneum is used to establish the surgical space [1], but it is undeniable that CO_2_ pneumoperitoneum could cause many physiological changes, resulting in compromised cardiopulmonary reserves, circulation, and internal environment unstability, especially in patients with impaired cardiopulmonary function [2–8]. In addition, there is still a potential risk of gas embolism [9–11]. These CO_2_ pneumoperitoneum associated complications mentioned above have driven the surgeons to explore another direction of laparoscopy, gasless laparoscopic surgery, mainly the abdominal wall lifting (AWL) method. Some clinical trials showed that the AWL method was associated with less pulmonary and hemodynamic changes, faster and more uneventful postoperative recovery [12–15], as well as more abdominal pain, worse exposure, and longer operation time [16–18].

With the intention of avoiding the adverse effects of CO_2_ pneumoperitoneum and retaining the mini-invasive characteristic of laparoscopic surgery at the same time, we designed a new gasless laparoscopic operation field formation (LOFF) device, which achieved success in large laboratory animal experiments [19] and its safety and efficiency were confirmed in clinical application for cholecystectomy [20]. The LOFF also obtained medical device registration certificate of the people’s republic of China (Registration Certificate No.: hxzz20192020273). Although the previous experiments and clinical application demonstrate its safety, efficiency, and advantages of maintaining the stability of circulation, respiratory function and acid–base equilibrium, evidence from prospective randomized clinical trials are still absent.

## Methods

### Trial design

We conducted a prospective, randomized, observer-blinded, single-center clinical trial comparing conventional CO_2_ pneumoperitoneum assisted laparoendoscopic single-site cholecystectomy (LESS) versus the gasless LOFF device assisted laparoendoscopic single-site cholecystectomy (LOFF-LESS). The study was performed with approval of the ethics committee of Shanghai East Hospital, Tongji University School of Medicine, Shanghai, China, and was registered on the Chinese Clinical Trial Registry website with the registration number of ChiCTR2000033702. Written informed consents were obtained from all patients who agreed to participate in this study voluntarily after having been informed of the objective, method, procedure, benefits, and risks of this study. Concealing the allocation for surgeons was unfeasible because of the different appearances of the LOFF device and the conventional port for single-incision laparoscopic surgery. Due to the same location and length of the transumbilical incision in both groups, patients and ward medical staff responsible for the postoperative care of the patients were blind to the allocation.

### Participants

Participants were recruited at Shanghai East Hospital, where both LESS and LOFF-LESS were performed routinely. The inclusion criteria were: aged more than 18 years; chronic cholecystitis complicated with cholecystolithiasis or gallbladder polyps diagnosed by ultrasound with indications for elective cholecystectomy; patients who were willing to be treated with single-incision laparoscopic cholecystectomy including conventional LESS and LOFF-LESS. Exclusion criteria included acute or subacute cholecystitis; patients with diagnosed or suspected choledocholithiasis, cholangitis, pancreatitis or malignancies; patients with a history of right upper abdominal surgery or suspected severe abdominal adhesion; umbilical hernia, urachal anomaly or previous transumbilical surgery; other acute or chronic diseases causing abdominal pain; pregnancy or lactation; American Society of Anesthesiologists (ASA) grade III, IV or V.

The sample size was calculated according to the respiratory parameters of LOFF-LESS and LESS procedures from our previous clinical study and experience before the initiation of this study. The estimated sample size was 50 patients per group with a risk of 0.05 and a power of 0.80. Eligible patients were randomly assigned in a 1:1 ratio to the LOFF-LESS and LESS groups. Randomization was performed using concealed opaque envelopes based on a computer-generated random allocation sequence before surgery.

### Procedures

All patients received the same and standardized preoperative management. Preoperative medication was antibiotic prophylaxis of intravenous cefoxitin sodium (2 g) 30 min before the operation. Upon arrival in the operating room, routine monitoring was applied, including circulatory and respiratory parameters after intubation. The anesthesia process was standardized, which was induced with intravenous injection of etomidate (0.3 mg/kg), sufentanil (0.3 µg/kg), and rocuronium (0.6 mg/kg). Intubation was performed and mechanical ventilation was set at a rate of 12 breaths/min and a tidal volume of 8 ml/kg. Anesthesia was maintained with continuous infusion of propofol (4 mg/kg/h) and remifentanil (0.04 ug/kg/min). Additional boluses of rocuronium were administered as required at the discretion of the clinician. To avoid possible confounding factors, no other adjustments to settings were performed under the premise of patient safety.

All the operations were performed by experienced surgeons, each of whom had carried out more than 1000 LESS operations and received special training of using the LOFF device with experience of over 100 LOFF-LESS operations. Both procedures began with the open method of a 20 mm vertical transumbilical incision down to the peritoneum. During laparoscopic procedure, all patients were placed in the head-up tilt position with the surgeons and the assistants standing at the left side of the patient and the monitor positioned to the right of the patient. In the LESS group, a disposable single-incision laparoscopic port (KangJi, Ltd, Hangzhou, China) with four working passages was inserted through the umbilical incision into the abdominal cavity. While in the LOFF-LESS procedure, the self-developed LOFF device, a triangular prism-shaped frame made of medical thermoplastic polyurethane (TPU) materials with a hollow passage for the entering of surgical instruments, was inserted into the abdominal cavity through the umbilical incision and sent to the anterior space of the liver, under the guidance of the laparoscope. The detailed instruction of using the LOFF device on cholecystectomy has been reported and the supplementary video has been deposited at http://links.lww.com/SLE/A243 [20]. After successful insertion, CO_2_ pneumoperitoneum was established to reach an intra-abdominal pressure (IAP) of 12 mmHg in the LESS procedure, in contrast, the IAP in the LOFF-LESS was zero. The surgical instruments and technique of removing the gallbladder were standardized and the same in both groups. The only difference between the two groups was the means of access. Conversion to open surgery or multi-port laparoscopic cholecystectomy (MPLC), which means the standard three-port laparoscopic cholecystectomy with an IAP of 12 mmHg, was done when believed to be necessary and the reasons for conversion were registered.

During the postoperative management, patients from both groups received a routine analgesic regimen of 30 mg intravenous ketorolac tromethamine. If the pain score was greater than 4, additional analgesic medication of 50 mg pethidine hydrochloride was injected intramuscularly and the time and frequency of administration were recorded. When patients complained of moderate-to-severe postoperative nausea and vomiting (PONV), 10 mg metoclopramide was injected intramuscularly. According to the department rules, the patients were discharged the second day after surgery, if there were no indications for continued hospitalization.

### Outcomes

The primary outcome was respiratory and hemodynamic parameters, continuously monitored during surgery. Mean arterial pressure (MAP) and end-tidal carbon dioxide (EtCO_2_) were recorded at the following time points: before skin incision (T_0_); 5, 15, 25, and 35 min after the establishment of CO_2_ pneumoperitoneum in the LESS or placement of gasless channel in the LOFF-LESS (T_1_, T_2_, T_3_, T_4_); 5 min after desufflation of CO_2_ pneumoperitoneum in the LESS or extubation of gasless chanel in the LOFF-LESS (T_5_).

Intra-operative unfavorable incidents, operation time, blood loss, Nassar operative difficulty grade [21], conversions to MPLC or open surgery, and extubation time were recorded. Operation time was noted from the skin incision to the end of skin closure. Extubation time was from the end of the operation to tracheal extubation. The measurement of pain was done 6 and 24 h after surgery, using a numeric rating scale (NRS) with scores ranging from 0 to 10. All patients in both groups received the same fixed postoperative medication, and additional medication for pain, PONV, and other symptoms were recorded. At 3 months follow-up, postoperative complications were also assessed.

### Statistical analysis

The statistical analyses were performed using SPSS for windows version 19.0 statistical software. The measurement data were described by mean (SD) and analyzed by *t* test. The categorical data were described as frequency (*n*) and analyzed with the chi-square test. One-way ANOVA for repeated measures was used to analyze changes over time within a group. Two-way ANOVA for repeated measures was used to verify differences both between groups and over time. *P* value less than 0.05 was considered statistically significant.

## Results

**Fig. 1:**
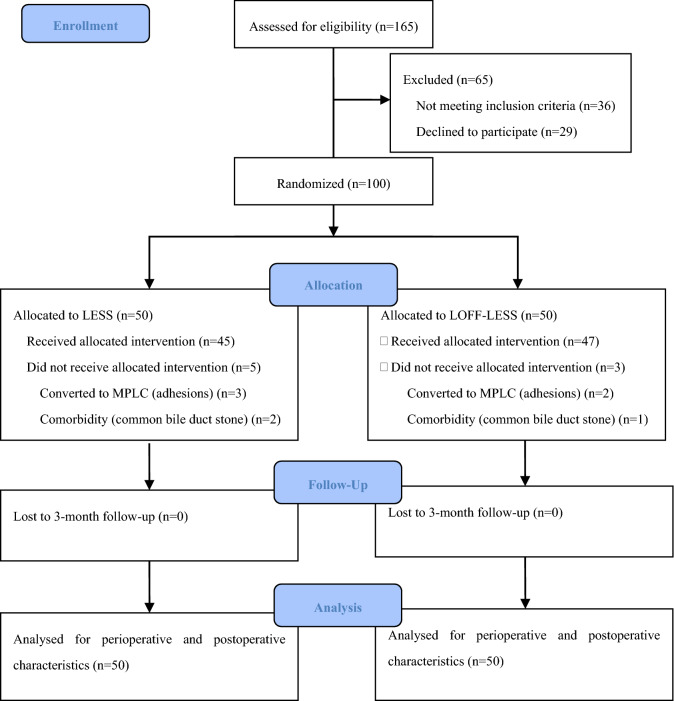
CONSORT flow diagram of patient assessment, randomization and follow-up

From June 2020, a total of 165 patients were assessed for eligibility of enrollment. 65 patients were excluded for the reasons of not meeting inclusion criteria and declined to participate. Randomization reached 100 patients by September 2020. Three patients were excluded after randomization for choledocholithiasis, and 5 patients were converted to MPLC (Fig. 1) (Supplementary Material: research dataset).

There were no statistically significant differences in baseline characteristics of age, sex, BMI, ASA score, Nassar operative difficulty grade, and the number of previous abdominal surgery between the two groups (Table 1).

**Table 1:** Baseline characteristics

Characteristics	LOFF-LESS (*n* = 50)	LESS (*n* = 50)	*P*
Age, years, mean (SD)	47.4 (13.3)	49.5 (13.9)	0.442
Sex ratio (female/male)	27/23	24/26	0.548
BMI (kg/m^2^), mean (SD)	23.5 (3.4)	23.2 (3.3)	0.597
ASA (I/II)	15/35	19/31	0.398
Previous abdominal surgery, *n* (%)	15 (30.0%)	15 (30.0%)	1.000
Nassar difficultly grade			0.678
I, *n* (%)	23 (46.0%)	25 (50.0%)	
II, *n* (%)	20 (40.0%)	16 (32.0%)	
III, *n* (%)	7 (14.0%)	9 (18.0%)	

Mean total operation times for the LESS and LOFF-LESS groups were 44.7 and 46.2 min respectively, and were comparable. Three patients from the LESS group and 2 patients from the LOFF-LESS group, with a Nassar difficulty grade of 3, were converted to MPLC, because of severe abdominal adhesion and difficult dissection of the Calot’s triangle, and the conversion rates were comparable. No patient in both groups had to be converted to open surgery. There was no patient experiencing remarkable blood loss (> 50 ml) during the surgery in both groups, and there were no serious intraoperative complications, including bile duct injury, hepatic injury, bowel injury, and vascular injury. The only intra-operative unfavorable event recorded was accidental gallbladder perforation, and the rates were similar in both groups (Table 2).

**Table 2:** Operative results

Results	LOFF-LESS (*n* = 50)	LESS (*n* = 50)	*P*
Total operation time, mean (SD)	46.2 (11.5)	44.7 (11.1)	0.502
Conversion to MPLC, *n* (%)	2 (4.0%)	3 (6.0%)	1.000^a^
Conversion to open surgery, *n* (%)	0 (0.0%)	0 (0.0%)	NA
Intra-operative severe complication, *n* (%)	0 (0.0%)	0 (0.0%)	NA
Gallbladder perforation, *n* (%)	6 (12.0%)	8 (16.0%)	0.564

^a^Indicated continuity correction

**Fig. 2:**
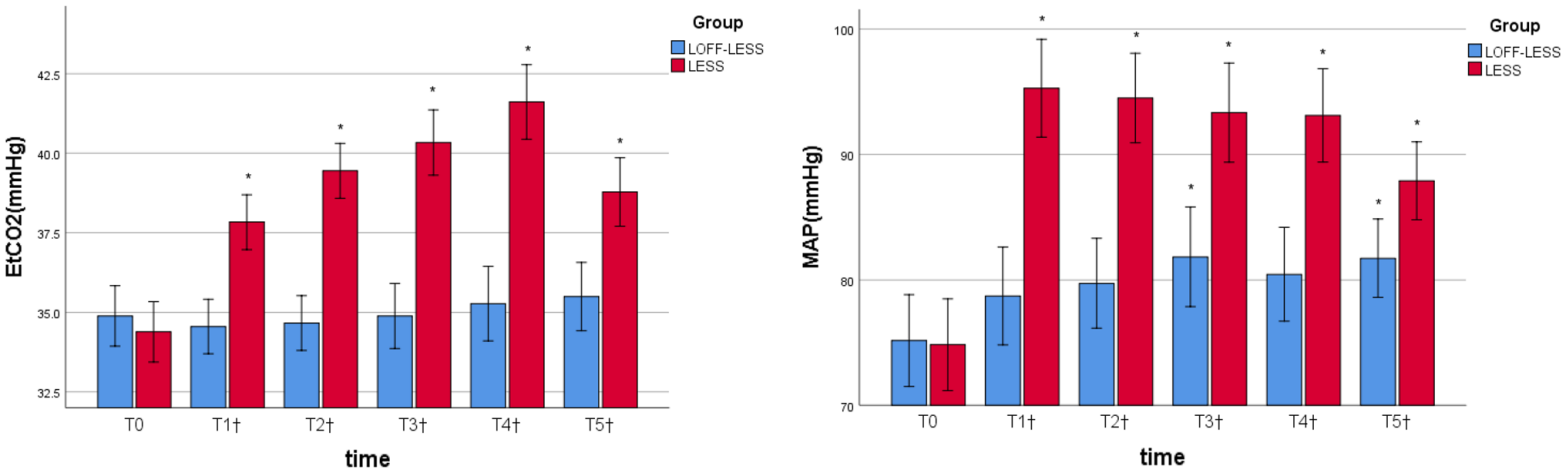
End-tidal carbon dioxide (EtCO_2_) and mean arterial pressure (MAP) at various times during operation in the LESS group and in the LOFF-LESS group. T_0_ = before skin incision; T_1_–T_4_ = 5, 15, 25 and 35 min after establishment of CO_2_ pneumoperitoneum in the LESS group and placement of gasless LOFF device in the LOFF-LESS group; T_5_ = 5 min after desufflation of CO_2_ in the LESS group and extubation of gasless LOFF device in the LOFF-LESS group. *P < 0.05 from baseline; ^†^*P* < 0.05 between groups. Error bars indicate standard error of the mean

Basal measurements of MAP and EtCO_2_ before skin incision (T_0_) showed no difference in both groups. Five minutes after the establishment of CO_2_ pneumoperitoneum or placement of gasless laparoscopic access (T_1_), a significant increase in MAP was noted in both groups, and the increase was significantly greater in the LESS group than in the LOFF-LESS group. The LESS group witnessed a significant upward trend in EtCO_2_ throughout the pneumoperitoneum procedure and a significant decrease in EtCO_2_ after desufflation of CO_2_. In general, compared to the LESS group, the values of MAP and EtCO_2_ in the LOFF-LESS procedure showed significantly less fluctuation (Fig. 2).

The mean tracheal extubation time in the LOFF-LESS group was shorter than it in the LESS group. There was no difference in pain scores 6 and 24 h postoperatively between the two groups. The number of patients needing additional drugs for PONV and postoperative pain was similar. It should be noted that 5 patients in the LESS group complained of discomfort of the precordial area and 2 of them required drugs to relieve precordial pain during 24 h after surgery, even though further blood tests and examination failed to confirm obvious abnormality. While no patients in the LOFF-LESS group complained of precordial discomfort. The duration of postoperative stay was similar between the 2 groups. During the 3 months follow-up, there were no severe postoperative complications including bile duct stenosis, residual stones in the common bile duct, incision hernia, and incision infection that occurred in both groups (Table 3).

**Table 3:** Postoperative characteristics

Results	LOFF-LESS (*n* = 50)	LESS (*n* = 50)	*P*
Tracheal extubation time (min), mean (SD)	4.9 (1.5)	6.4 (2.8)	0.001
Postoperative pain score (NRS), mean (SD)			
At 6 h	3.1 (0.8)	3.2 (0.9)	0.554
At 24 h	2.0 (0.9)	2.1 (0.8)	0.396
Patients needing additional medication for PONV, *n* (%)	6 (12.0%)	7 (14.0%)	0.766
Patients needing additional analgesics for pain, *n* (%)	2 (4.0%)	4 (8.0%)	0.674^a^
Patients with precordial discomfort, *n* (%)	0 (0.0%)	5 (10.0%)	0.056^b^
Postoperative severe complication	0 (0.0%)	0 (0.0%)	NA
Postoperative hospital stay	2.0 (0.1)	2.1 (0.3)	0.172

^a^Indicated continuity correction

^b^Indicated Fisher’s exact test

## Discussion

The aim of this study is to verify the safety and efficacy of the new gasless LOFF device on laparoendoscopic single-site cholecystectomy. Meanwhile, we designed this trial to explore the possible superiority of the new gasless LOFF-assisted LESS to conventional LESS in maintaining intraoperative respiratory and hemodynamic stability and avoiding CO_2_ pneumoperitoneum-associated complications.

Our study did not find any difference in operation time, conversion rate, blood loss, intraoperative unfavorable incidents, hospital stay, postoperative pain, and postoperative severe complication between the two groups, and the outcomes of both groups are also comparable to the other reported data [22, 23], which demonstrated the safety and effectiveness of the new LOFF-LESS method. However, due to the fact that all the patients included in this study underwent strict screening with low anesthetic risk and simple condition, the current evidence could only confirm the safety of the new LOFF device applied in simple cholecystectomy, referring to patients meeting the inclusion and exclusion criteria, mainly elective cholecystectomy without choledocholithiasis, cholangitis, pancreatitis, malignancies, pregnancy, and right upper abdominal surgery or suspected severe abdominal adhesion. And all the enrolled patients were operated on by surgeons with specific training and profound experience of using this new LOFF device, meanwhile, this new technique is not familiar to the majority of surgeons, so the results of this study are valid specifically within the constraints of this study, and special training is required before using this LOFF device. In this study the total operation time of both groups was comparable, but according to the consultant’s experience, an advantage of the new gasless procedure is that the hollow passage of the device allows different surgical instruments entering the passage and reaching the operative field at the same time, saving the time spent on exchanging surgical instruments. There was no difference in postoperative pain scores between the two groups, which was in accordance with the fact that the range and length of the transumbilical incision were the same in both groups. Because the minimum length of hospital stay was limited to at least 2 days according to the department routine, it cannot be ruled out that one of the two procedures might shorten hospitalization.

Our study demonstrated that the LOFF-LESS procedure was associated with more stable intra-operative hemodynamic and respiratory changes. Compared with the LOFF-LESS group, intra-operative MAP and EtCO_2_ in the LESS group were significantly higher and showed greater dramatic variation, which was in accordance with reported data [24, 25]. During pneumoperitoneum, carbon dioxide was continuously insufflated into the abdominal cavity to maintain an IAP of 12 mmHg, which can compress the abdominal aorta and peripheral blood vessels, thus increasing mean arterial pressure, systemic and pulmonary vascular resistances, and decreasing cardiac index [26]. Some changes of the humoral factors induced by increased IAP, such as an increase in plasma vasopressin level, might also contribute to these hemodynamic changes [27]. And CO_2_ absorption through the abdominal cavity could lead to acidification of the interior surface of the abdominal cavity, increase the CO_2_ level in blood, with a potential of systemic acidosis. To maintain body fluid acid–base balance, extra work must be done by the respiratory system to remove the increased CO_2_ in blood. Not only the absorption of CO_2_ will increase the risk of acidosis, but also the increased IAP will compress the lung mechanically, further increasing respiratory and metabolic burden. Although most of these values retained to normal in a short time after surgery, the establishment of CO_2_ pneumoperitoneum does exert adverse effects on cardiovascular and pulmonary functions and increases the physiological burden. Thus there is significance to maintaining intra-operative cardiovascular and pulmonary stability.

This study also showed that postoperative recovery after the gasless procedure was faster and uneventful when CO_2_ pneumoperitoneum was not established. The LOFF-LESS group had a shorter tracheal extubation time. CO_2_ retention might explain the delayed extubation in the LESS group. Stable hemodynamic and pulmonary parameters in the gasless group might also improve the patients’ postoperative comfort level, and further reducing the recovery time after anesthesia. Another unexpected finding was that the LESS procedure might be associated with postoperative cardiac symptoms, while no patients in the LOFF-LESS group complained of that, which might be attributable to the side effects of CO_2_ pneumoperitoneum on cardiopulmonary function [28].

The study had some limitations. First, all cholecytectomies in this study were restricted to simple cholecystectomies. Therefore, to further verify the results and support the superiority of the LOFF device in patients with cardiopulmonary insufficiency, a stratified study with a larger number of patients including patients with cardiopulmonary diseases from multi-centers was required. Second, since the LOFF device was a newly developed medical instrument and all the enrolled patients were essentially interested in the new technique, which biases the secondary outcomes, including postoperative pain and discomfort towards the new device, the external validity of this study might be limited. Therefore, further research using the complete blind method is required to reduce this kind of bias. Third, although this study found that the LOFF-LESS procedure was associated with faster and uneventful postoperative recovery, the specific mechanism needs to be further explored. Fourth, our study did not confirm the long-term effects of the LESS and LOFF-LESS methods, so that further follow-up is warranted. In addition, we plan to further explore the potential predominance of the LOFF-LESS method in patients with impaired cardiopulmonary function to identify the practical beneficiaries of the new gasless method.

## Conclusion

In conclusion, with adequate surgeon training and well patient selection, the newly developed gasless LOFF device for laparoendoscopic single-site cholecystectomy is safe and feasible. The new gasless method also avoids CO_2_ pneumoperitoneum associated complications, providing a smoother operative course and a faster and uneventful postoperative recovery. But considering the not yet perfect design of the LOFF device and its shortcomings in complicated surgeries, currently, the new LOFF device could only be considered as an alternative to the conventional laparoscopic port for simple cholecystectomies without complications such as choledocholithiasis, cholangitis, pancreatitis, and severe abdominal adhesion, mainly for patients with cardiopulmonary insufficiency to prevent the potential side effects of CO_2_ pnuemoperitoneum.

## Supplementary Information

Below is the link to the electronic supplementary material.

Supplementary file1 (XLSX 19 KB)

## Data Availability

The datasets generated during this study are included in this article and its supplementary information files.
